# Diagnosis and Management of Cystic Lesions of the Pancreas

**DOI:** 10.1155/2011/478913

**Published:** 2011-08-22

**Authors:** Niraj Jani, Murad Bani Hani, Richard D. Schulick, Ralph H. Hruban, Steven C. Cunningham

**Affiliations:** ^1^Department of Medicine, Saint Agnes Hospital, Baltimore, MD 21229, USA; ^2^Department of Surgery, Saint Agnes Hospital, Baltimore, MD 21229, USA; ^3^Department of Surgery, The Sol Goldman Pancreatic Cancer Research Center, The Johns Hopkins Hospital, Baltimore, MD 21287, USA; ^4^Department of Pathology, The Sol Goldman Pancreatic Cancer Research Center, The Johns Hopkins Hospital, Baltimore, MD 21231, USA

## Abstract

Pancreatic cysts are challenging lesions to diagnose and to treat. Determining which of the five most common diagnoses—pancreatic pseudocyst, serous cystic neoplasm (SCN), solid pseudopapillary neoplasm (SPN), mucinous cystic neoplasm (MCN), and intraductal mucinous papillary neoplasm (IPMN)—is likely the correct one requires the careful integration of many historical, radiographic, laboratory, and other factors, and management is markedly different depending on the type of cystic lesion of the pancreas. Pseudocysts are generally distinguishable based on historical, clinical and radiographic characteristics, and among the others, the most important differentiation is between the mucin-producing MCN and IPMN (high risk for cancer) versus the serous SCN and SPN (low risk for cancer). EUS with FNA and cyst-fluid analysis will continue to play an important role in diagnosis. Among mucinous lesions, those that require treatment (resection currently) are any MCN, any MD IPMN, and BD IPMN larger than 3 cm, symptomatic, or with an associated mass, with the understanding that SCN or pseudocysts may be removed inadvertently due to diagnostic inaccuracy, and that a certain proportion of SPN will indeed be malignant at the time of removal. The role of ethanol ablation is under investigation as an alternative to resection in selected patients.

## 1. Introduction

Pancreatic cysts are common in the general population. The reported incidence of asymptomatic cysts varies widely, largely due to differences in study design, ranging between 0.7% and 24.3% [1–4]. The lowest estimate comes from a study employing both single- and multidetector CT scanners and relying on original dictated reports as opposed to rereview of images [1], while the highest estimates come from autopsy studies and studies including both symptomatic and asymptomatic patients [3, 4]. The incidence of truly asymptomatic cysts in the general population is approximately 2.6% [2]. In large series of pancreatic cysts [5], most (71%) cysts are largely asymptomatic and range from benign to premalignant to malignant cysts. The most useful first dichotomy in the long differential diagnosis (Table 1) of pancreatic cysts is their classification as either neoplastic or nonneoplastic. Nonneoplastic cysts include pseudocysts, retention cysts, and duplication cysts, whereas neoplastic cysts are further broadly classified as mucinous and nonmucinous cysts. The more common—and more commonly malignant—mucinous neoplasms include primarily intraductal papillary mucinous neoplasm (IMPN) and mucinous cystic neoplasms (MCN), while nonmucinous neoplastic cysts include primarily serous cystic neoplasm (SCN), solid pseudopapillary neoplasm (SPN), and usually solid neoplasms with degenerative cystic changes [6, 7]. Whereas most serous cystic neoplasms are not malignant, intraductal papillary mucinous neoplasms and mucinous cystic neoplasms can harbor an associated invasive carcinoma and should be treated as having malignant potential. 

Differentiating among these cysts is challenging, and a variety of modalities—including imaging, cytology, and cyst fluid analysis—are useful. The management of pancreatic cystic lesions continues to evolve. The purpose of this paper is to review the current approaches to the diagnosis and management of pancreatic cystic lesions.

## 2. Nonneoplastic Pancreatic Cysts

Pseudocysts are defined as a collection of pancreatic fluid enclosed by a wall of nonepithelialized granulation tissue (Figure 5: Pancreatic pseudocyst). They are caused by the abnormal release of pancreatic enzymes into the tissues that might result from pancreatic duct disruption related to pancreatitis or trauma. In the absence of a history of pancreatitis or trauma, this diagnosis should be considered very unlikely. 

Retention cysts, duplication cysts, and other rare nonneoplastic cysts of the pancreas (Table 1) can be difficult to distinguish from more common lesions, and therefore clinical, laboratory, and radiographic characteristics guide the decision to treat or to observe, as discussed below.

## 3. Neoplastic Pancreatic Cysts

The most important distinction among neoplastic cysts is the categorization of mucinous versus nonmucinous. The most common nonmucinous neoplastic cysts are SCN and SPN, while the most common mucinous lesions include IPMN and MCN.

SCNs represent approximately 7%–36% of all cystic neoplasms [5, 8, 9] and are present in middle-aged females, evenly distributed throughout the pancreas, and characterized grossly by a microcystic appearance and a central stellate scar that often corresponds radiographically with a pattern of central sunburst calcification on CT imaging (Figure 6: SCN). They grow slowly, and their potential for malignancy is extremely low, but when these cysts are greater than 4 cm or causing symptoms, surgical resection is recommended [10, 11].

Previously known by the eponymous terms Hamoudi tumor or Franz tumor, SPNs are typically benign mixed solid/cystic tumors that are associated with young age (median 32–38 years) and female gender (84%–89%) [12, 13]. Grossly, they are often filled with bloody or necrotic debris and radiographically have a similarly mixed solid/cystic appearance, with calcifications commonly seen (Figure 7: SPN) [14]. SPNs are now considered potentially malignant, and 10% to 15% of patients have or ultimately develop metastases [13, 15–17]. 

Cystic variants of solid tumors are some of the many rare cystic lesions that may also be present in the pancreas. For example, ductal adenocarcinoma, acinar adenocarcinoma, and neuroendocrine tumors all may undergo cystic degeneration and may present as primarily cystic lesions (Table 1) [5, 7, 8, 18]. In a recent study of over 1,400 cystic lesions of the pancreas, 7% were cystic pancreatic neuroendocrine tumors and 14% were adenocarcinomas with cystic degeneration [8]. 

Previously known as “mucin-producing tumor” and “mucinous ductal ectasia,” IPMN is a grossly visible (typically ≥1.0 cm) intraductal epithelial neoplasm composed of mucin-producing cells. IPMNs may arise from either the main pancreatic duct (MD IPMN), branch ducts (BD IPMN), or both (Figures 2 and 3) [19, 20]. IPMN is most common in elderly patients, males more than females, and located in the head of the pancreas more often than the tail. The malignant potential is variable, depending predominantly on the location of the IPMN: the percentage of IPMNs found after resection to harbor a malignancy (invasive carcinoma or carcinoma in situ) ranges in various studies from 6% to 46% for BD IPMN and from 49% to 92% for MD IPMNs [5, 8, 21–26]. If carcinoma develops within an IPMN, survival depends on the subtype: colloid adenocarcinomas are associated with a more favorable survival than tubular adenocarcinomas, which are associated with a 5-year survival rate not statistically different from conventional, non-IPMN-related pancreatic ductal adenocarcinoma [27, 28].

In contradistinction to IPMN, MCNs do not involve the duct system and have an associated ovarian-type of stroma. MCNs also have a strong female predominance and are found almost exclusively in the body and tail of the pancreas. MCNs are typically macrocystic (>2 cm), spheroid, solitary, and associated with a normal pancreatic duct with which there is no communication [7] (Figure 4: MCN). In a recent, large, two-institution series, approximately 11% were invasive [23].

## 4. Diagnostic Modalities

The two noninvasive imaging modalities which have been most frequently used to evaluate pancreatic cysts are computed tomography (CT) and magnetic resonance imaging (MRI). Pancreas-protocol CT scan (with the IV contrast bolus timed for both arterial and venous phases and typically with water as the oral contrast to minimize artifacts arising from denser contrast media) has become the preferred modality to evaluate the pancreas due to its ease, relatively low expense, and diagnostic accuracy [29–31]. However, some authors have argued that MRI with magnetic resonance cholangiopancreatography (MRCP) is the best noninvasive method for identifying the presence or absence of communication between pancreatic cysts and the pancreatic ductal system [32]. While MRI/MRCP has the clear advantage over CT of not involving the use of ionizing radiation, it lacks the ability to sample cyst fluid for analysis, which, as discussed below, can help distinguish between high-risk mucinous and low-risk nonmucinous cysts. 

Endoscopic retrograde cholangiopancreatography (ERCP) has been useful in cases of IPMN especially when combined with pancreatoscopy and/or intraductal ultrasound. Hara et al. [33] found that lesions protruding more than 4 mm into the pancreatic duct were malignant in 88% of cases. ERCP, although more invasive than MRCP, is very useful in defining the communication of the cyst with the main pancreatic duct and provides another method for tissue acquisition. Due to its associated risk of pancreatitis, however, and the improving quality of MRCP, its role has become limited in favor of endoscopic ultrasound (EUS).

EUS with fine-needle aspiration (FNA) has been extensively studied in the detection, diagnosis, and treatment of pancreatic cysts [34, 35]. The diagnostic accuracy of EUS morphology alone is widely variable with the largest prospective study reporting an accuracy of 50% for identification of macrocystic septations or adjacent mass [36]. FNA increases the sensitivity of EUS by allowing for cyst fluid analysis and cytology to further differentiate mucinous cysts, serous cysts, and pseudocysts [7]. Columnar epithelial cells which stain for mucin are characteristic of MCN and IPMN, whereas cuboidal cells which stain for glycogen are associated with serous cystadenomas. A recent study by Rogart et al. [37] showed that EUS with FNA and cyst wall puncture (passing the needle repeatedly through the far wall of the cyst to obtain wall epithelium for cytology after simple aspiration of cyst fluid) increased the cytologic yield by 37% compared to simple FNA with fluid analysis alone. The increased diagnostic capabilities of EUS/FNA compared with noninvasive modalities must be balanced, however, with the associated risks, including pancreatitis and hemorrhage [37]. In general EUS/FNA is associated with a favorable risk/benefit ratio, as evidenced by several large studies including several hundred patients suffering no major complications [34, 35]. In addition to being used to evaluate cystic lesions of the pancreas found by other modalities, EUS/FNA is also potentially useful as a screening modality in individuals deemed to have ≥10-fold increased risk of harboring a pancreatic cancer, such as those with ≥3 relatives with pancreas cancer in the same lineage [38].

While imaging modalities provide morphologic characteristics of the lesions, it is the fluid analysis and tumor-marker levels that ultimately classify a cyst as of high or low risk for malignancy. Cyst fluid amylase level is elevated in pseudocysts and IPMNs. IPMNs have elevated amylase levels because, by definition, they involve the pancreatic duct system. By contrast, SCNs and MCNs, because they do not communicate with the pancreatic duct system, typically have lower amylase levels. Carcinoembryonic antigen (CEA) level is the most studied and widely used tumor marker in differentiating mucinous from nonmucinous lesions. Although no cutoff level is universally agreed upon, a cyst-fluid CEA level <30 ng/mL has a sensitivity of 79% and specificity of 73% in differentiating nonmucinous from mucinous lesions [39]. In the Cooperative Pancreatic Cyst Study, a higher cut-off (192 ng/mL) was found to be optimal, with a sensitivity of 73%, a specificity of 84%, and an accuracy of 79% for diagnosing nonmucinous from mucinous lesions [36]. Although CEA has been found to have the highest accuracy among cyst fluid analyses in distinguishing mucinous from nonmucinous cysts, other tumor markers have also been predictive. For instance, in the same Cooperative Study, cyst fluid CA19-9 levels had a sensitivity of 68%, a specificity of 62%, and an accuracy of 66% (*P* = 0.004) with a cutoff value of 2900 U/mL for differentiating nonmucinous from mucinous lesions [36]; corresponding numbers for CA72-4 were 80%, 61%, and 72% (*P* = 0.001).

The demographic, historical, radiographic, gross, and cyst fluid analysis characteristics described above are summarized in Table 2 and are electronically available in an interactive, online pancreatic cyst worksheet available at http://pathology.jhu.edu/pancreas/professionals/ipmn.php [7].

### 4.1. Emerging Modalities

Loss-of-heterozygosity studies and DNA mutational analysis of cyst fluid have shown that the presence of a point mutation in the *KRAS* gene is 96% specific in detecting a mucinous neoplasm, and when there is a *KRAS* gene point mutation coupled with allelic loss at selected markers, there is a 96% specificity in detecting malignancy (invasive versus in situ carcinoma not specified) [41]. However, this study has been rightfully criticized for harboring a selection bias resulting from the exclusion of nonoperated patients from cyst fluid DNA analysis [42]. Furthermore, there is poor correlation between cysts with high CEA levels and those with *KRAS* point mutations and allelic loss. There are a number of other biomarkers that are currently under evaluation to predict risk in pancreatic cysts [43, 44].

Confocal laser endomicroscopy (CLE) [45] is an exciting emerging diagnostic modality that employs a low-power laser to illuminate tissue with subsequent detection light reflected from the tissue through a small probe (pCLE) or needle (nCLE). It is “confocal” because both illumination and collection systems are aligned in the same focal plane [45]. While largely still experimental, nCLE has been used successfully in a porcine model to collect real-time, in vivo pancreatic images at histologic resolutions and of acceptable image quality [46].

## 5. Management

### 5.1. Nonneoplastic Cysts

Initially, the management of pseudocysts is conservative since as many as 60% may completely resolve spontaneously within a year [47]. As such, surveillance is the first-line therapy for noninfected pancreatic pseudocysts and may be done with US, CT, or MRI. Pseudocysts that either cause severe symptoms or are large and refractory to surveillance should be drained percutaneously, endoscopically, or surgically. The disadvantages of percutaneous drainage include risk of infection, fistula formation, and a low rate (21%) of resolution [48]. Open and laparoscopic internal surgical management—including internal and external drainage as well as resection—is effective, but is associated with 12%–35% complication rate, including hemorrhage, infection, and fistulae, and a mortality rate of 1% [49–51]. Endoscopic drainage has been reported to achieve a similarly high success rate but with lower rates of complications, including bleeding, infection, perforation, and mild pancreatitis, which is generally self-limited [52, 53]. Endoscopic drainage has therefore become the preferred modality for draining cysts which have a mature wall and are within 1 cm of the gastrointestinal lumen. In a recent large retrospective study by Ahn et al. [54], single-step EUS-guided transmural drainage and stent placement was effective in 89% of patients with complete drainage, with an overall recurrence rate of 12% and minor complications in 11% of patients. 

Unlike pancreatic pseudocysts, which are typically identifiable as such based on historical, clinical, laboratory, and radiographic information, other nonneoplastic cysts such as duplication cysts, retention cysts, congenital epithelial cysts, and lymphoepithelial cysts are rarer, and not easily diagnosed preoperatively. As such, they are typically subjected to the various diagnostic modalities described above in an effort to classify them correctly as low-risk versus high-risk cysts and they are treated or surveilled accordingly.

### 5.2. Neoplastic Cysts

#### 5.2.1. Indications for Resection

In the absence of randomized controlled data to guide treatment recommendations, the Sendai International Consensus Guidelines [21, 55], first published online in 2005 by the International Association of Pancreatology, identified several factors as relative indications for resection of IPMN. These include a main-duct component, diameter >3 cm, any solid component, and the presence of symptoms attributable to the cyst, such as abdominal pain, weight loss, and pancreatitis (Table 2). Rapid rate of growth of the cyst and young age (such that life-long surveillance would be prohibitively burdensome for the patient) may be considered relative indications outside the Sendai Guidelines. Resection recommended as the mainstay of treatment for lesions thought to have increased the potential for harboring significant dysplasia or an associated invasive carcinoma, and indeed it is the only potentially curative option for such lesions. 

Unlike a cystic lesion thought to be IPMN, which may be observed or resected, depending on the above-mentioned risk factors, any lesion thought to be MCN should be resected until data are available to better stratify these patients, if such data ever exist. 

Regarding the serous lesions SCN and SPN, all lesions known to be SCN may be left in place and all those known to be SPN should be resected. In reality, however, a given cystic lesion of the pancreas is not generally known to be one or the other with sufficient certainty, even despite all of the above-discussed diagnostic modalities. Therefore, each pancreatologist and patient must together carefully weigh the risks and benefits of resection and surveillance on a case-by-case basis (see Surveillance, below).

The chief difficulty is, of course, the fact that the only way to achieve a definitive diagnosis in many cases of pancreatic cysts is to remove the cyst and subject it to pathologic evaluation. Although pancreatectomy is curative in most cases of cystic lesions of the pancreas, it is associated with a perioperative morbidity rate of 30–60% [56–58] and a mortality rate ranging from <1% to 2% [56–59]. In addition to complications associated with any operation in general, such as bleeding and infection, complications specific to the resection of pancreatic lesions include pancreatic or biliary fistula, delayed gastric emptying, and pancreatic insufficiency, both exocrine and endocrine.

#### 5.2.2. Cyst Ablation

**Figure 1:**
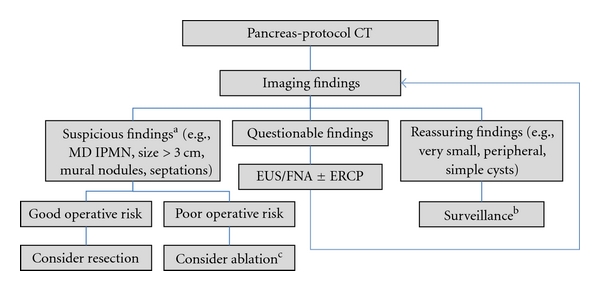
Pancreatic cyst therapeutic algorithm.  ^a^Also considered are nonimaging findings such as symptoms attributable to the cyst, rapid growth, and young age. ^b^Surveillance may be performed initially at close intervals (e.g., 3 mo), and later spaced out to every 6, 12, or 24 months. ^c^NB: cyst ablation is largely experimental and not appropriate for main-duct IPMNs. Abbreviations: see text.

**Figure 2:**
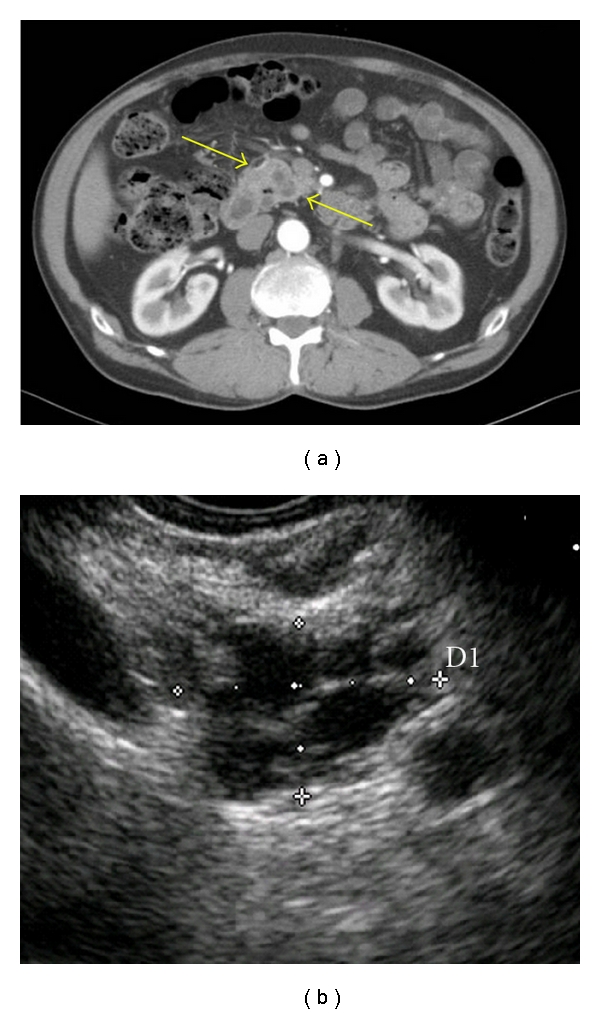
Main-duct intraductal papillary mucinous neoplasm. (a) Typical CT (arrows) and (b) EUS (cross marks) appearance.

**Figure 3:**
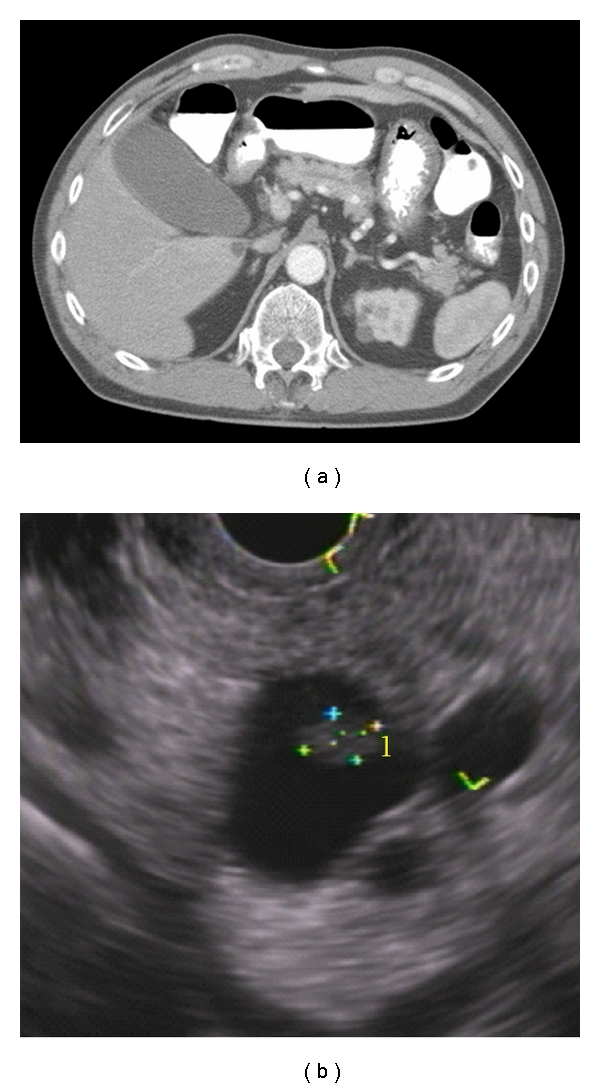
Branch-duct intraductal papillary mucinous neoplasm. (a) CT and (b) EUS showing associated mass (cross marks).

**Figure 4:**
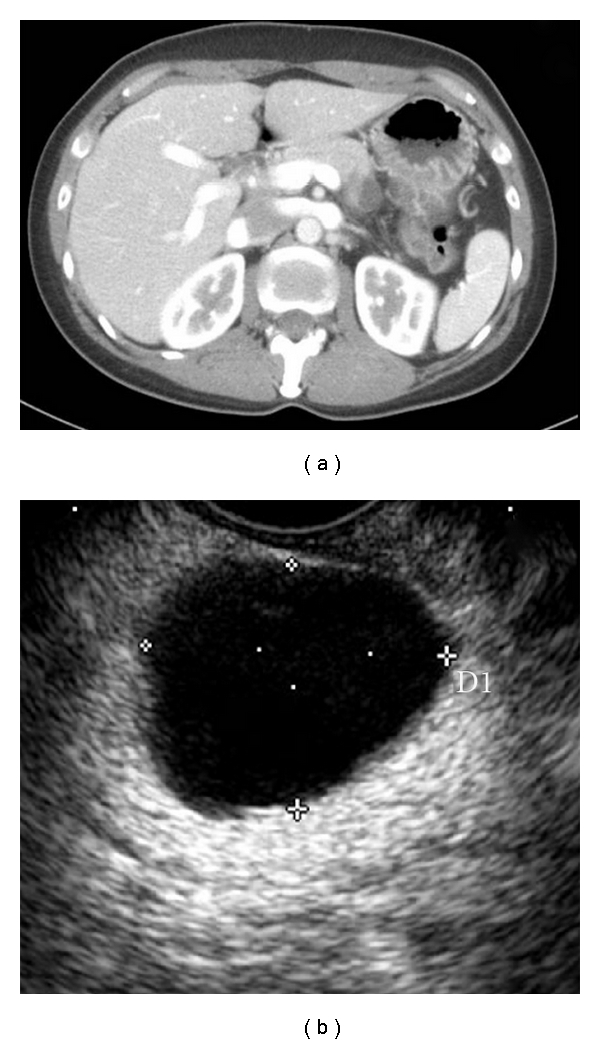
Mucinous cystic neoplasm. (a) Typical CT and (b) EUS appearance of a well-rounded hypodense and anechoic, respectively, pancreatic cyst in the tail of the gland of a female patient.

**Figure 5:**
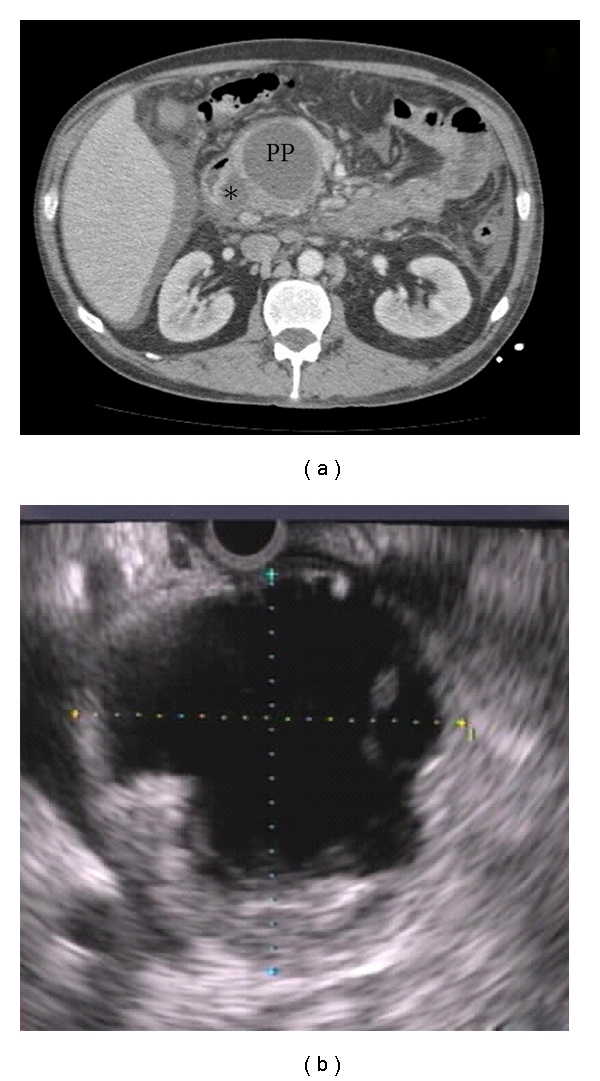
Pancreatic pseudocyst. (a) Typical appearance on CT, showing a dominant pancreatic pseudocyst (PP) with a smaller pseudocyst (asterisk) impinging slightly on the air- and fluid-filled duodenum. (b) Typical EUS appearance of a pseudocyst, with debris.

**Figure 6:**
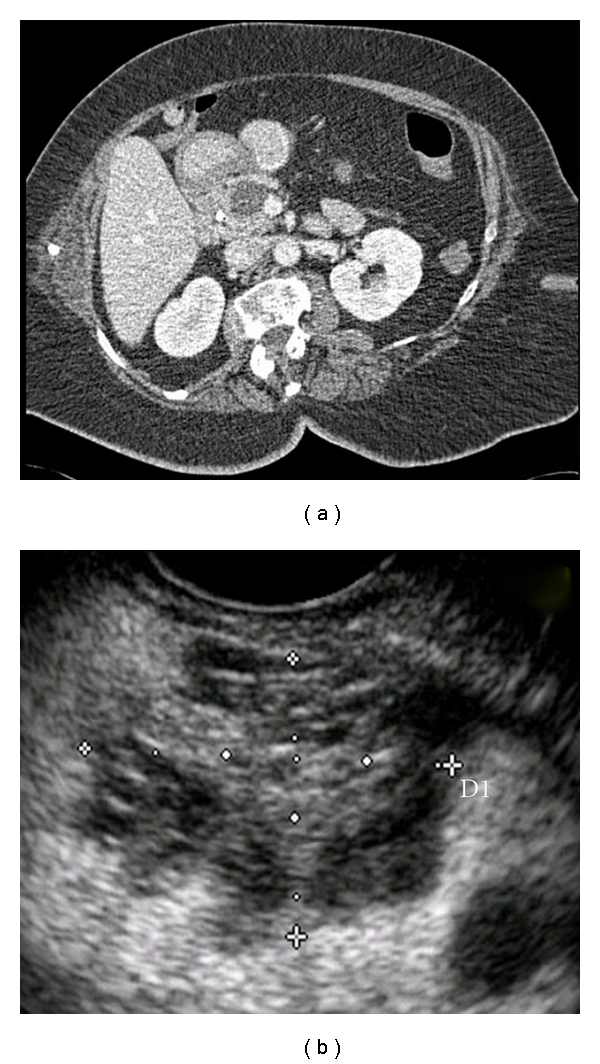
Serous cystic neoplasm: (a) CT and (b) EUS images both showing the central starburst calcification pattern characteristic of serous cystic neoplasms.

**Figure 7:**
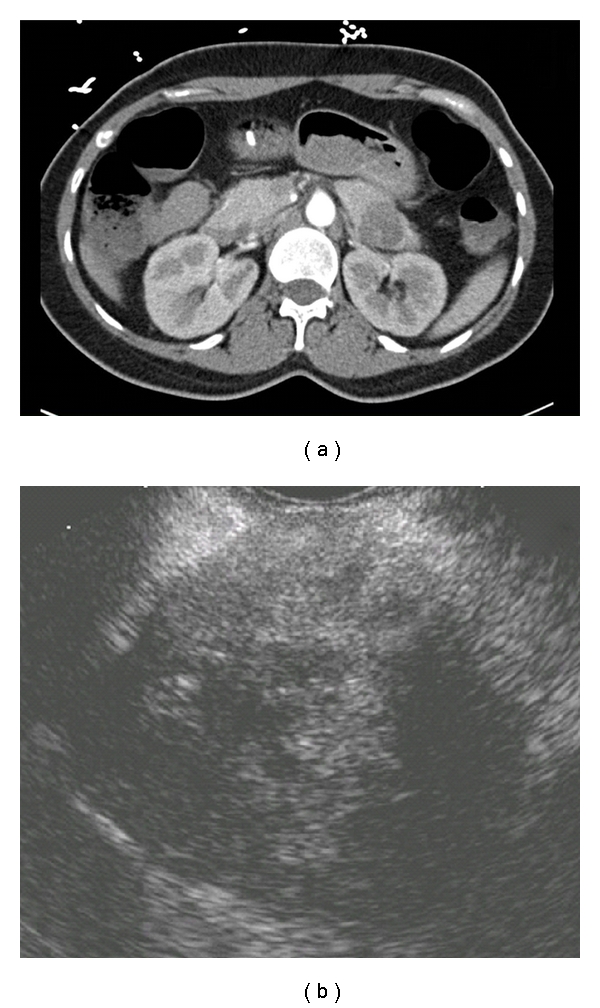
Solid pseudopapillary neoplasm: (a) Typical CT and (b) EUS appearance of solid and cystic components.

In an effort to avoid a more invasive treatment and the associated complications, pancreatic cyst ablation has been suggested both as an experimental approach to treatment for pancreatic cysts in general and for treatment of those patients specifically deemed unfit or at a too high risk for a major operation (Figure 1) [60].

Although less commonly employed than resection, pancreatic cyst ablation is an increasingly studied modality, typically using EUS to guide injection of alcohol or other ablative agents into the cyst cavity. Ethanol has the advantages of being safe, inexpensive, readily available, and having the potential to rapidly ablate the entire cyst wall epithelium. A 2005 pilot study using escalating doses (5% to 80%) of ethanol for 3- to 5-minute lavage [61] showed histological evidence of epithelial ablation in resected cysts. Patients reported no symptoms at 2 hours, 72 hours, and 6–12 months following the procedure, with no complications detected [61], although theoretical complications include acute pancreatitis, hemorrhage, intoxication, and abdominal pain.

In an effort to assess effectiveness as well as to further assess safety, DeWitt et al. [62] compared ethanol ablation to saline lavage in a randomized controlled trial including 42 ethanol-lavaged and 17 saline-lavaged patients. Ethanol lavage resulted in greater decrease in pancreatic cyst size (−43%), compared with saline (−11%), with similar safety profile [62]. Four patients underwent resection after lavage of mucinous cysts (2 who decided to drop out after lack of response (one to saline and one to ethanol) and 2 whose cyst fluid had atypical cells), and histology of resected pancreata showed IPMNs in 3 and MCN in 1 patient; not surprisingly, there was more extensive ablation (50% to 100% of cyst epithelium) in the ethanol group than the saline group (0%). Resolution by CT imaging was seen in 33% (12 of 36 cysts lavaged with saline alone [1], ethanol alone [4], saline then ethanol [13], ethanol then ethanol [18]) [62].

Subsequent studies of EUS with ethanol ablation have expanded the field to include ablation of septated cysts (successful) [63], the addition of paclitaxil to increase ablative capacity of the lavage (62% of patients had complete resolution) [64], and longer (2 years) followup of ablated patients (no recurrence during second year) [65]. Although these preliminary data suggested that ethanol ablation is safe and feasible, prospective randomized trials with longer followup in more patients comparing ablation with resection are needed.

#### 5.2.3. Surveillance

Patients too unfit to undergo resection or whose cysts do not meet the above-mentioned Sendai criteria for treatment may undergo surveillance (Figure 1). Indeed, not only a patient's physiologic fitness for resection but also a patient's goals must be considered. To this end, the Markov modeling and nomograms have been used in a recent study [66] to assist patients with small asymptomatic BD IPMNs with decision making regarding the risks and benefits of resection versus surveillance. The decision to resect or to surveil depended on the patient's age and comorbidities, the size of the cyst, and whether the patient values quality or quantity of life more; that is, overall survival versus quality-adjusted survival [66]: those valuing primarily survival, irrespective of quality of life, would benefit most from resection of lesions >2 cm. However, for patients valuing quality of life over longevity, a 3-cm threshold for resection would be more appropriate.

## 6. Summary

The diagnosis and management of cystic lesions of the pancreas is challenging and continues to evolve. The five most common diagnoses are pseudocysts, SCN, SPN, MCN, and IPMN. Pseudocysts are generally distinguishable based on historical, clinical, and radiographic characteristics, leaving the most important differentiation being between the mucin-producing (often malignant or premalignant), MCN and IPMN, and the serous (generally benign), SCN and SPN, cysts. EUS and FNA with cyst-fluid analysis have an increasingly important role in diagnosis. Among mucinous lesions, those that require treatment (resection currently) are any MCN, any MD IPMN, and BD IPMN larger than 3 cm, symptomatic, or with an associated mass. In the future, ethanol ablation may well supplant resection or at least provide an alternative treatment in selected patients.

## Figures and Tables

**Table 1:** Differential diagnosis of pancreatic cysts.

Nonneoplastic lesions	Neoplastic lesions
	IPMN
Pseudocysts	MCN
	SCN
Syndromes causing multiple cysts	SPN
(i) Autosomal dominant polycystic disease	
(ii) Cystic fibrosis	

Infectious cysts	Cystic variants of solid tumors
(i) Hydatid cysts	(i) Cystic teratoma
(ii) Abscess	(ii) Cystic ductal adenocarcinoma
	(iii) Cystic neuroendocrine tumor
	(iv) Cystic acinar cell carcinoma
Lymphoepithelial cysts	(v) Cystic metastases
Congenital epithelial cysts	
Duplication cysts	
Retention cysts	

**Table 2:** Distinguishing features of pancreatic cystic lesions*.

Typical characteristics	IPMN	MCN	SCN	PSEUDO	SPN	LEC	cNET	cPDAC
Age group	Elderly	Middle	Middle-elderly	Any	Young	Elderly	Middle-Elderly	Elderly
Gender	>50% male	95% female	>50% female	>50% male	80%–90% female	80% male	50% each	>50% male
History	Asx; pain; ± jaundice	Asx; Pain; nausea	Asx; VHL	Pancreatitis	Asx; pain; nausea	Asx	Asx; Fxnl; MEN	Asx; pain; ± jaundice
% of all cysts***	17%–40%	9%–28%	7%–36%	1%–19%	1%–13%	<2%	<8%	13%–16%

Location in pancreas	Head in 70%; multifocal	Body/Tail in 95%	Anywhere	Anywhere	Anywhere	Peripheral	Anywhere	Anywhere
Shape	Ovoid	Spheroid	Ovoid	Spheroid	Ovoid	Ovoid	Spheroid	Variable
Locularity	Any	Uni- or oligo-	Oligo- or multi-	Uni-	Oligo- or Multi-	Oligo-	Uni-	Any
Duct com-munication	Common	No	No	Common	No	No	No	Some
Calcification	No	No	Central sunburst	No	Some	No	Some	No

Cyst fluid appearance	Viscous, clear, muc.	Viscous, clear, muc.	Thin, clear, nonmuc.	Opaque, bloody/ necrotic debris	Opaque, bloody/ necrotic debris	Nonmuc., crystalline debris	Nonmuc.	Thin
High CEA/Mucin**	**+**	**+**	**−**	**−**	**−**	**−**	**−**	** ±**
High Ca19-9	** ±**	** ±**	**−**	**−**	**−**	**−**	**−**	** ±**
High amylase	**+**	**−**	**−**	**+**	**−**	**−**	**−**	** ±**

Epithelium	Columnar, papillary	Columnar	Cuboidal	No epithelium	Poorly cohesive cells with nuclear grooves	Squamoid	Uniform	Gland-forming
Stroma	Fibrotic	Ovarian	Fibrotic	Fibrotic	Sometimes hyalinized	Lymphoid	Sometimes hyalinized	Fibrotic

Abbreviations: IPMN: intraductal papillary mucinous neoplasm; MCN: mucinous cystic neoplasm; SC: serous cystadenoma; PSEUDO: pancreatic pseudocyst; SPN: solid-pseudopapillary neoplasm; LEC: lymphoepithelial cyst; cNET: cystic neuroendocrine tumor; cPDAC: pancreatic ductal adenocarcinoma with cystic degeneration; VHL: von Hippel-Lindau disease; muc.: mucinous; Nonmuc: nonmucinous; Asx: asymptomatic; Fxnl: functional.

***Percentages references [8, 9, 22, 40].

**May be positive in cases of luminal contamination of endoscopic needle aspirate.

NB: These data are derived generalizations of the literature, with the understanding that there is significant overlap among cyst types and there are inherent sampling errors associated with various tests; diagnostic and treatment decisions should not rely solely on the information presented in this paper.

*Table modified from [7] by Cunningham et al. Intraductal papillary mucinous neoplasms are differentiated from other pancreatic cystic lesions. World J Gastrointest Surg 2010; 2(10): 331–336. An electronic worksheet version of this table is available at http://pathology.jhu.edu/pancreas/professionals/ipmn.php.
